# Leadership Amongst Regional and National Surgical Organizations: The Tides Are Changing

**DOI:** 10.7759/cureus.10827

**Published:** 2020-10-06

**Authors:** Stephanie M Krise, Ian Etheart, Adam Perzynski, John Como, Mary Carneval, Kristen Conrad-Schnetz

**Affiliations:** 1 Surgery, Ohio University Heritage College of Osteopathic Medicine, Cleveland, USA; 2 Surgery, West Virginia School of Osteopathic Medicine, Lewisburg, USA; 3 Statistics, MetroHealth Medical Center, Cleveland, USA; 4 Trauma, MetroHealth Medical Center, Cleveland, USA; 5 Surgery, Cleveland Clinic Euclid Hospital, Cleveland, USA; 6 Surgery, Cleveland Clinic South Pointe Hospital, Cleveland, USA

**Keywords:** gender equity, academic leadership, academic surgery, surgical careers, gender parity, academic promotion, surgical organization, organization leadership, women in leadership, women in surgery

## Abstract

Introduction: Leadership amongst professional organizations is a key opportunity for scholarly activity which is essential for academic advancement. Our objective was to examine the differences between men and women in leadership within surgical organizations.

Methods: Credentials were obtained through an internet search. Variables included organization type, leadership role, gender, advanced degree, medical school graduation year, and publications. A bivariate analysis was performed between genders. A p-value <0.05 was considered statistically significant.

Results: Five hundred forty-three leaders were identified in 43 surgical organizations. There was a significant difference in the number of male and female leaders (72.7% vs 27.3%, p=0.016). Women were most likely to hold the role of “Other”, which consisted of lower-level leadership roles including committee chair positions and resident and medical student delegates (35.5%). Fewer women had publications (85.8% vs 92.9%, p=0.01), more women had advanced degrees (24.5% vs 17.0%, p=0.049), and women were involved earlier in their careers (5.9 years, 95% CI 4.1-7.7 years, p<0.001) than their male colleagues.

Conclusion: Gender disparity in leadership of surgical organizations exists. Women are involved earlier in their careers and hold lower-level leadership positions reflecting potential for increased involvement in high-level leadership roles in the future. Data need to be trended to discern if women in surgical organizations rise within leadership roles as more women continue to enter surgical subspecialties.

## Introduction

Prior studies have shown women who graduate from U.S medical schools have been more likely to pursue academic medicine than men, but significantly fewer advance from Assistant Professor to Associate and Full Professors [1]. In most academic institutions, ranks of Associate and Full Professors come with tenure, compared to Assistant Professorships which are entry-level positions. From 1994 to 2015, the number of female Full Professors of Surgery increased by 366.2%, but women still only constituted 9.8% of all Full Professors of Surgery [2]. The study concluded that, at this rate, it would take an estimated 121 years for gender parity to be reached [2].

Scholarly activity and leadership are essential for attaining advancement in clinical academia. Recent research on the attributes of academic surgeons found dissemination of knowledge, publications, and leadership in professional organizations to be defining characteristics amongst those considered academic surgeons [3]. Much literature is available on gender differences in research funding and publication rates in surgical subspecialties, but little data exist reviewing gender equity in leadership amongst surgical organizations [4,5].

Examination of gender equity in leadership of surgical organizations is important given the gender disparity amongst Full Professors of Surgery and the need for leadership in pursuit of academic promotion. Our objective was to examine the differences between men and women in leadership within surgical organizations. We hypothesize there will be a gender disparity among high-level leadership of surgical organizations, including the roles of President, President-Elect, and Past-President, without a similar disparity in credentials.

## Materials and methods

In July 2018, 43 regional and national surgical organizations were examined in a cross-sectional study of gender equity in leadership positions. Data was collected through an internet search of surgical organizations, their leaders, and leadership credentials. Google was used to search surgical organization websites for biographical information and/or curriculum vitiate of the organization’s current leaders. A secondary search was then completed for remaining information using professional and clinical/hospital websites and professional networking sites. PubMed was used for publication data. Variables collected included gender, year of medical school graduation, organization subspecialty, leadership role, fellowship training, advanced degree, employment at an academic institution, and publication status. Gender was delineated as male/man or female/woman. Non-binary classification was not included.

Organizations were chosen by selecting osteopathic surgical organizations and matching their allopathic counterparts. The Association of Women Surgeons was included to avoid misrepresenting the number of women in leadership by excluding a group comprised of mostly women. Organizations were varied in size and subspecialty as well as documented as acute care/trauma, burn, colorectal, otolaryngology (ENT), general, hepato-pancreato-biliary, obstetrics and gynecology (OB/GYN), orthopedic, ophthalmology, otolaryngology, pediatric, plastics and reconstructive, surgical endoscopy, thoracic, transplant, urological, vascular, and other surgical organizations. “Other” surgical organizations comprised organizations that represented a multitude of subspecialties (American College of Surgeons, American College of Osteopathic Surgeons, Association of Women Surgeons, etc.).

Leadership roles were categorized as President, Past-President, President-Elect, Vice President, Secretary/Treasurer, Board of Governor/Council Member, and Other. The role of “Other” consisted of committee chair members and medical student and resident delegates. High-level leadership was considered roles of President, Past-President, or President-Elect. The roles in the category of “Other” were considered low-level leadership roles. Advanced degrees documented were Master of Science, Master of Public Health, Master of Business Administration, Doctor of Philosophy, and Other. Leaders with two or more advanced degrees as well as those with any degree other than those previously listed were categorized as “Other.” 

Leaders who held multiple roles within a single organization were only counted once with their highest leadership role documented. If leaders held positions in multiple organizations, all leadership roles were documented.

A bivariate analysis was performed to evaluate the difference between men and women in leadership roles, publication status, advanced degrees, and medical school graduation year. A chi-square test was used for the difference of two proportions. A p-value of <0.05 was considered statistically significant.

## Results

**Figure 1:**
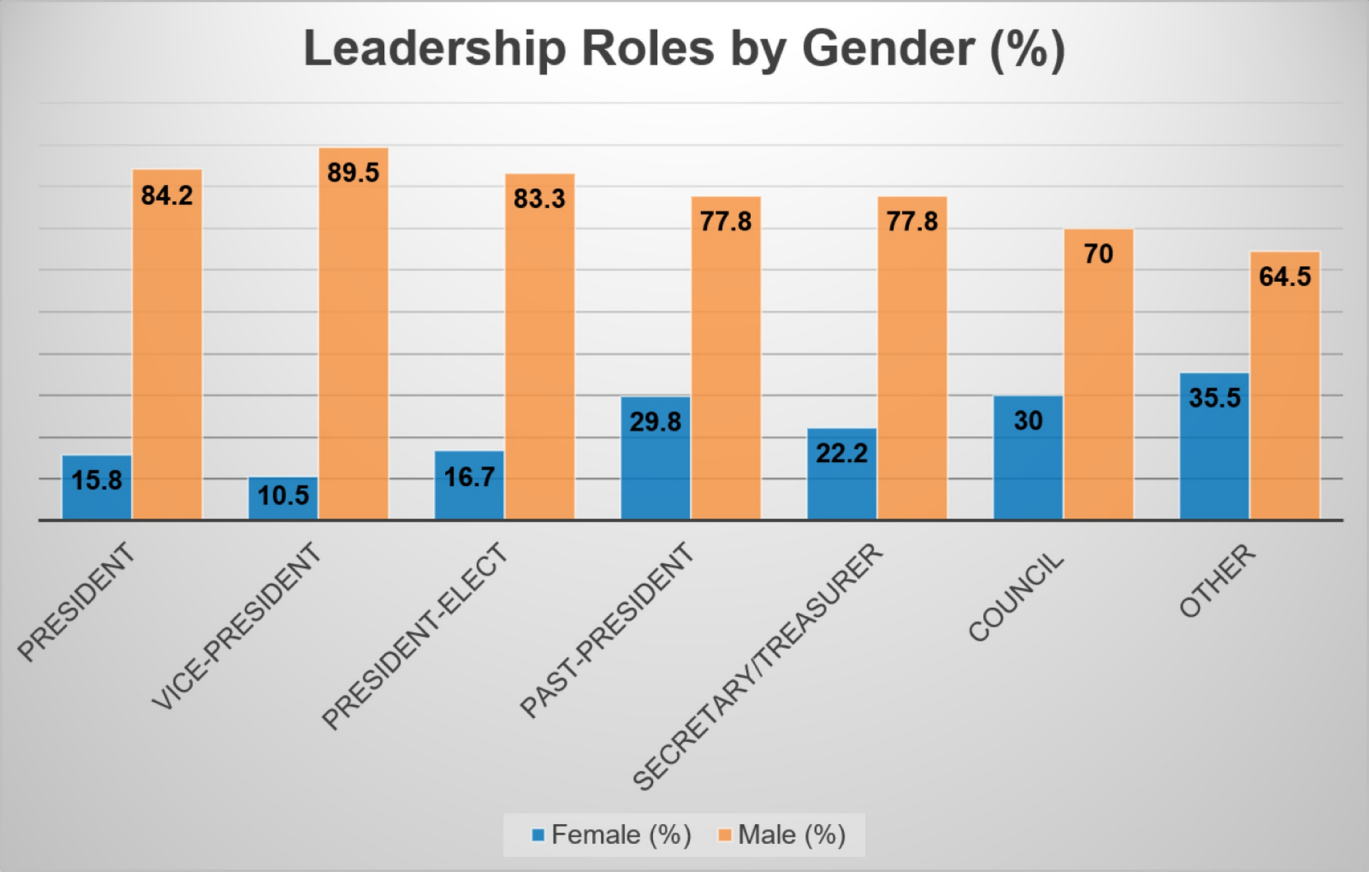
Leadership roles by gender

Five hundred forty-three leaders were identified within 43 organizations (Table 1). One hundred forty-eight (27.3%) were women and 395 (72.7%) were men, showing a statistically significant gender difference in leadership (p=0.016). Six (15.8%) women held the role of President compared to 32 men (84.2%). Women were least likely to be Vice-President, with only four (10.5%) holding the position compared to 34 (89.5%) men (Figure 1). The leadership position with the highest percentage of women (35.5%) was the role of “Other” (Figure 1).

**Table 1:** Surgical organizations listed alphabetically

Surgical Organizations Alphabetically	
American Academy of Ophthalmology	American Surgical Association
American Academy of Orthopedic Surgeons	American Urological Association
American Academy of Orthopedic Surgeons	Americas HepatoPancreatoBiliary Association
American Association for Hand Surgery	Association for Academic Surgery
American Association for the Surgery of Trauma	Association of Women Surgeons
American Association for Thoracic Surgery	Central Surgical Association
American Association of Genitourinary Surgeons	Eastern Association for Surgery of Trauma
American Association of Hip and Knee Surgeons	Orthopedic Trauma Association
American Association of Plastic Surgeons	Plastic Surgery Research Council
American Association of Transplantation	Society for Basic Urologic Research
American Burn Association	Society for Surgery of Alimentary Tract
American College of Obstetrics and Gynecology	Society for Vascular Surgeons
American College of Osteopathic Surgeons	Society of American Gastrointestinal and Endoscopic Surgeons
American College of Surgeons	Society of Thoracic Surgeons
American Council of Academic Plastic Surgeons	Society of University Surgeons
American Orthopedic Association	Southeastern Society of Plastic and Reconstructive Surgeons
American Osteopathic Academy of Orthopedics	Southern Association for Vascular Surgery
American Osteopathic Colleges of Ophthalmology and Otolaryngology	Southern Surgical Association
American Pediatric Surgical Association	Southern Thoracic Surgical Association
American Society of Breast Surgeons	Southwestern Surgical Congress
American Society of Colon and Rectal Surgeons	The American Osteopathic College of Proctology
American Society of Transplant Surgeons	

Women had a higher percentage of advanced degrees than men (24.5% vs 17.0%, p=0.049). Women were most likely to have a Master of Public Health (38.9%) and men were most likely to have a Doctor of Philosophy (32.8%). There was also a significant difference between women and men who had been published (85.8% vs 92.9%, p=0.01) (Figure 3). Approximately half of osteopathic surgeons had publications compared to allopathic surgeons (52.5% vs. 95.6%, p=<0.00001). Of published surgeons, there was a difference between allopathic men and women (97.2% vs 91.7%, p=0.009) and osteopathic men and women (59.1% vs 33.3%, p=0.084). 

**Figure 2:**
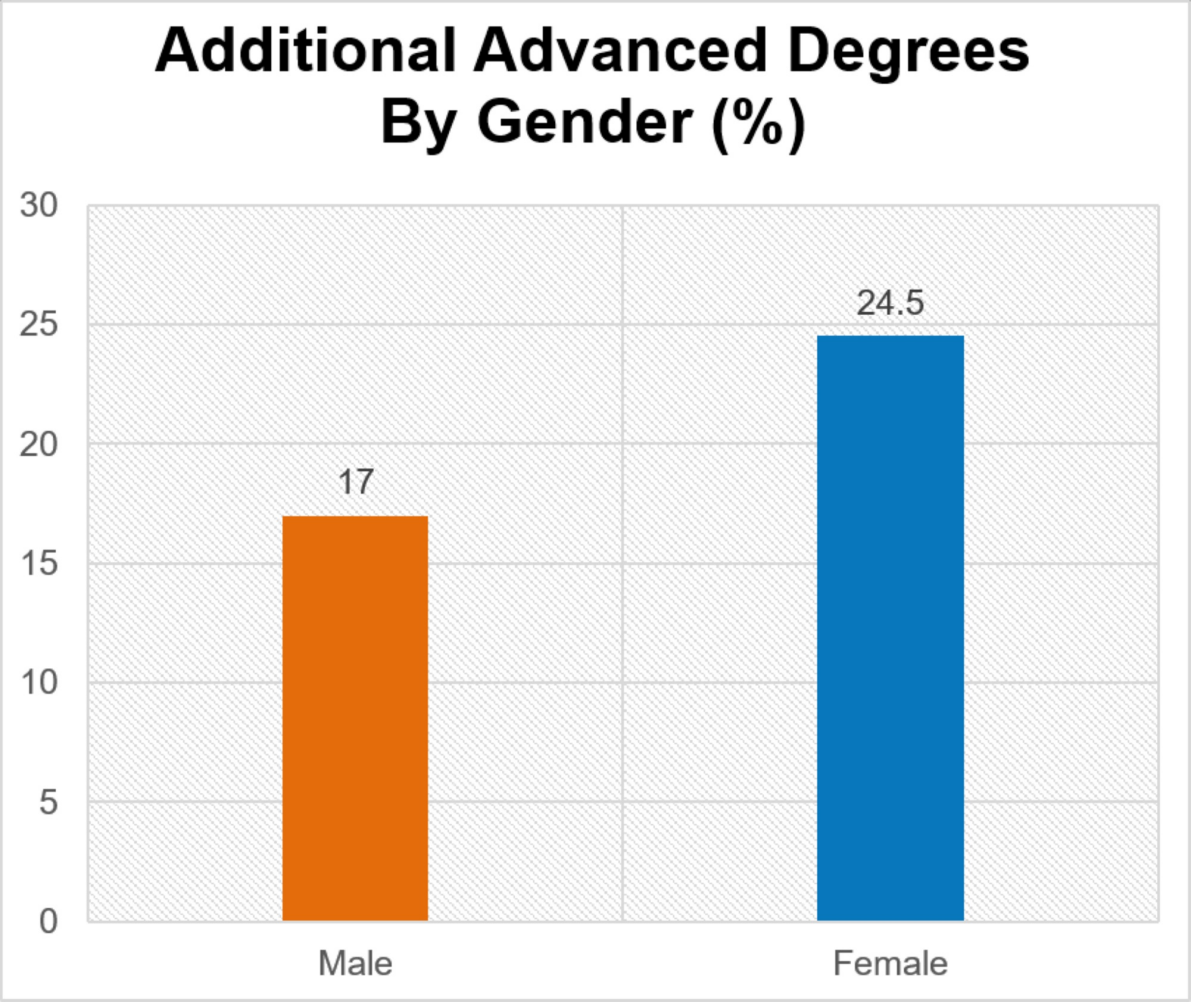
Advanced degree by gender

**Figure 3:**
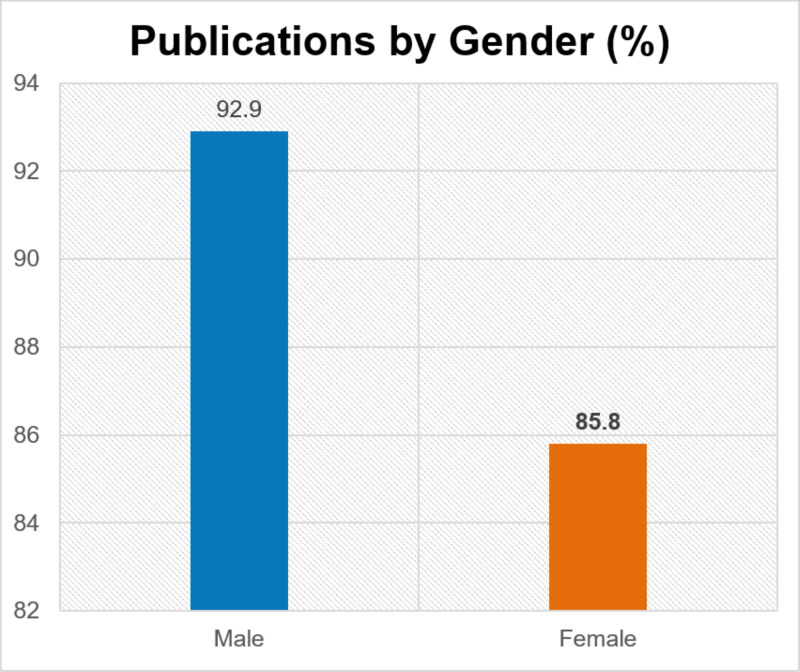
Publications by gender

The average time from medical school graduation for women leaders was 25.16 years. This was statistically significant (5.9 years, p<0.001, 95% CI 4.1-7.7 years) compared to men at 31.06 years. 

**Figure 4:**
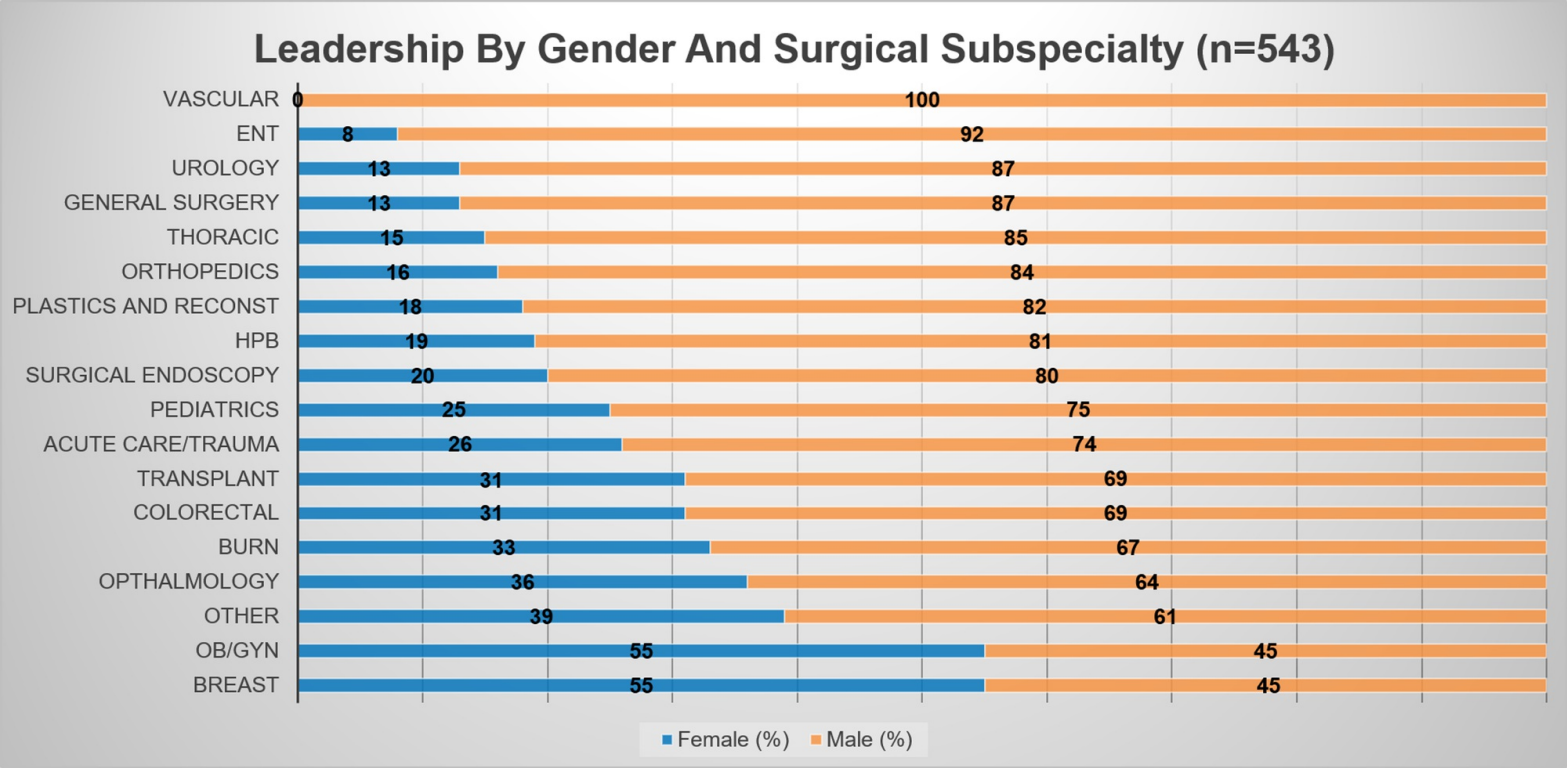
Leadership by gender and subspecialty ENT- Otolaryngologic surgery, HPB- Hepato-pancreato-biliary surgery, OB/GYN- obstetrics and gynecological surgery

When comparing types of organizations, OB/GYN and breast surgery had the highest percentage of women leaders (54.5%), and vascular surgery had the least, with no leadership positions held by women (Figure 4). Eleven of 18 surgical subspecialties identified had women in high-level leadership roles of President, Past-President, or President-Elect.

## Discussion

In 2017, 20.6% of practicing general surgeons in the United States were women, yet women comprised only 13.3% of all general surgery leadership positions. Of all surgical subspecialties included, OB/GYN had the highest number of women practicing at 57% and held the highest number of leadership positions at 54.5%. Women held 0% of leadership positions in vascular surgery, although 13.1% of vascular surgeons were women. However, thoracic, orthopedic, ophthalmic, plastic, and urological surgery all had a higher percentage of women in leadership than in practice [6]. While women are proportionally represented in some surgical subspecialties, our study shows the leadership roles held by women were most likely to be lower-level roles including committee chair, medical student, and resident delegates. Although women were most likely to hold these lower-level roles, still only 35.5% of these roles were held by women compared to 64.5% of these roles held by men.

The trend of fewer women in leadership roles and those women holding lower-ranking roles parallels the gender disparity in academic surgery, even though our data show women were found to have more advanced degrees than men. This suggests there is a systemic bias that exists not only in academia, but also within surgical organizations. It is possible that women may be gaining additional advanced degrees to compensate for their lack of leadership and publication rates when applying for academic positions or possibly pursuing these degrees in hope of becoming more qualified for leadership positions.

Our data show that more women have advanced degrees than men, but fewer women have publications. Previous research from 2004 showed that women in general surgery have one-third to one-half as many journal articles as men [7], and there is documented gender disparity in National Institutes of Health (NIH) grant funding in ophthalmology and otolaryngology [4,5]. Mitigating the disparity of awards granted to female researchers may assist in continued research output and increased publication rates by women, and furthermore increase leadership opportunity in surgical organizations and advancement in academia [4,5,7]. It is also worth noting our data show osteopathic surgeons were considerably less likely to be published than allopathic surgeons. This could be due to different attitudes on publications and leadership qualities between the osteopathic and allopathic organizations or due to difference in grant funding and research opportunities.

Historically, women have been under-represented in medicine and even more so in surgical subspecialties [8]. For the last 10 years, the number of women matriculating into medical school has been increasing, and 2017-2018 marked the first year that greater than 50% of matriculants to allopathic medical schools were women [9]. Over the last 10 years there has also been an increase in the number of women matching into surgical subspecialties [6,10]. As of 2017, women comprised over one-third of Accreditation Council of Graduate Medical Education (ACGME) residents in surgical subspecialties, increased from one quarter in 2007 [6,10].

As an increasing number of women advance into surgical subspecialties, we expect the rate at which they join professional organizations and embrace leadership responsibilities will follow suit. However, traditional female gender roles and responsibilities within families may still be impacting women’s decision or ability to engage in leadership and furthermore, academia. In a nationwide postal survey of physicians from 2010 to 2011, women were more likely than men to have spouses or domestic partners who were employed full time (85.6% vs 44.9%) and spent an average of 8.5 more hours per week on domestic activity. Women were also more likely to take time off for disruptions in childcare [11]. It is unclear how this will change as the traditional family paradigm shifts and more women in the United States continue to enroll in medical school and pursue surgical fields. Our results show that women in our study held lower ranking leadership positions and were involved nearly six years earlier in their career compared to their male colleagues. The authors feel women’s earlier involvement and lower-level representation in organizations presents women with an opportunity for advancement to higher-level leadership roles in the future. Providing mentorship and sponsorship to these women may result in increased leadership opportunities within these organizations.

Role models, mentors, work/life balance, and family planning have been cited as helping women make the decision to pursue a surgical field and affect career planning [8,12,13]. In accordance, there should be continued emphasis on mentoring women in surgery throughout their careers and in their leadership endeavors. The Association of Women Surgeons offers mentoring and support for women in all stages of their careers including Professional Development Coaching and a Gender Equity Toolkit that provides resources on the gender salary gap in surgery [14]. The Association of American Medical Colleges’ Group on Women in Medicine and Science (GWIMS) works to address gender equity, recruitment, retention, and career advancement for women within academic medicine [15]. GWIMS also provides a toolkit designed to address topics relevant to women in academic medicine, as well as a task force with national resources to assist in dismantling barriers to women in medicine and science [15]. These resources should be taken advantage of by surgical organizations and incorporated into their own culture. The American College of Surgeons has already taken steps toward including specific networking and mentoring resources for women by their creation of the Women in Surgery Committee (WiSC) [16]. It is our hope that organizations creating and using these, or similar tools, will facilitate surgical organizations to include more women in high-level leadership roles in the future.

Leadership in professional surgical organizations is one modality for advancement in academic surgery. In addition to scholarly activity, leadership within organizations provides networking opportunities and exposure to potential referees when applying for academic advancement. Given this relationship, gender parity within surgical organizational leadership is important for women to continue to advance in academic surgery. This data should be revisited to identify trends of advancement if in leadership roles held by women, closing the gender equity gap in leadership amongst surgical organizations.

Our study was limited by not assessing all surgical organizations within the United States and therefore not accounting for all leadership positions. Additionally, we did not collect data on membership demographics of each organization to assess whether leadership was proportional to its membership regarding gender. Leader credentials were collected via internet search of organizational and institutional websites and were not all self-reported. We were not able to account for name changes and are therefore not able to recognize if publications from previously listed names were excluded. 

## Conclusions

Women continue to be under-represented in leadership roles of surgical organizations at all levels of leadership. While there are many qualities that go into acquiring academic leadership, publications and leadership have been found to be important credentials. We have found that there continues to be gender disparity in publication rates despite women having higher rates of additional advanced degrees. The authors feel further inquiry should be obtained regarding the influence of advanced degree type on leadership status. Women participating at an earlier stage of their career and in lower-ranking positions may elucidate a transformation occurring and surgical organizations should take advantage of the resources available to continue to encourage and mentor women to pursue leadership roles. Surgical organization leadership should be re-examined in the future to identify whether women continue to increase their engagement in leadership and if those holding lower-level roles advance to higher levels within their respective organizations.
